# A total crapshoot? Evaluating bioinformatic decisions in animal diet metabarcoding analyses

**DOI:** 10.1002/ece3.6594

**Published:** 2020-07-23

**Authors:** Devon R. O'Rourke, Nicholas A. Bokulich, Michelle A. Jusino, Matthew D. MacManes, Jeffrey T. Foster

**Affiliations:** ^1^ Department of Molecular, Cellular, and Biomedical Sciences University of New Hampshire Durham NH USA; ^2^ Pathogen and Microbiome Institute Northern Arizona University Flagstaff AZ USA; ^3^ Laboratory of Food Systems Biotechnology Institute of Food, Nutrition, and Health ETH Zurich Zurich Switzerland; ^4^ Biology Department William & Mary Williamsburg VA USA; ^5^ Center for Forest Mycology Research USDA Forest Service Northern Research Station Madison USA; ^6^ Department of Biological Sciences Northern Arizona University Flagstaff AZ USA

## Abstract

Metabarcoding studies provide a powerful approach to estimate the diversity and abundance of organisms in mixed communities in nature. While strategies exist for optimizing sample and sequence library preparation, best practices for bioinformatic processing of amplicon sequence data are lacking in animal diet studies. Here we evaluate how decisions made in core bioinformatic processes, including sequence filtering, database design, and classification, can influence animal metabarcoding results. We show that denoising methods have lower error rates compared to traditional clustering methods, although these differences are largely mitigated by removing low‐abundance sequence variants. We also found that available reference datasets from GenBank and BOLD for the animal marker gene cytochrome oxidase I (COI) can be complementary, and we discuss methods to improve existing databases to include versioned releases. Taxonomic classification methods can dramatically affect results. For example, the commonly used Barcode of Life Database (BOLD) Classification API assigned fewer names to samples from order through species levels using both a mock community and bat guano samples compared to all other classifiers (vsearch‐SINTAX and q2‐feature‐classifier's BLAST + LCA, VSEARCH + LCA, and Naive Bayes classifiers). The lack of consensus on bioinformatics best practices limits comparisons among studies and may introduce biases. Our work suggests that biological mock communities offer a useful standard to evaluate the myriad computational decisions impacting animal metabarcoding accuracy. Further, these comparisons highlight the need for continual evaluations as new tools are adopted to ensure that the inferences drawn reflect meaningful biology instead of digital artifacts.

## Graphical abstract

Modern animal diet metabarcoding experiments are complicated assortments of many alternative bioinformatic pipelines. We chose to investigate how parameter choices in three core aspects common to any diet analysis (clustering/denoising, classification, and database development) may influence subsequent inferences. Our findings outline both a set of recommended processes and a template for future evaluations as new bioinformatic tools become available.

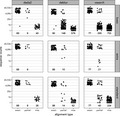

## 1 INTRODUCTION

Metabarcoding of animal diets has fundamentally changed our insights into what species are eating, expanding our understanding of dietary diversity and food web complexity (Clare, 2014; Pompanon et al., 2012; Symondson, 2002; Valentini, Pompanon, & Taberlet, 2009). The factors needed to be considered for amplicon analyses are extensive, including sample collection, primer and barcode design, tag jumping, sequencing platform, as well as sequence data processing and taxonomic assignment (reviewed by Alberdi, Aizpurua, Gilbert, & Bohmann, 2018; Clare, 2014; Pompanon et al., 2012; Schnell, Bohmann, & Gilbert, 2015). While modern sequencing approaches have enabled a broad range of studies, this expansion has resulted in myriad customized molecular and bioinformatics workflows, making comparisons among studies difficult. While past reviews have explored how wet‐bench decisions and sequencing platforms influence diet interpretations (Alberdi et al., 2018; Alberdi et al., 2019; Palmer, Jusino, Banik, & Lindner, 2018), many computational decisions essential to any metabarcoding analysis have yet to be thoroughly compared. This work sheds light on several common yet often overlooked and undocumented bioinformatics steps in metabarcoding studies to illustrate which processes most influence interpretations of animal diets.

Molecular metabarcoding experiments typically seek to characterize the composition of a community, but analytical decision‐making is a complicated process. Establishing best practices for sequence filtering and classification are frequently determined using mock communities—samples with known sequence identity and abundance. This practice is commonplace in microbial gene marker research (Bokulich et al., 2016), but less common for arthropod datasets (Braukmann et al., 2019). Mock communities can be used to assess systematic error and biases in observed sequence data (Gohl et al., 2016), optimize filtering parameters (Bokulich et al., 2013), understand tradeoffs among sequence error correction approaches (Nearing, Douglas, Comeau, & Langille, 2018), and evaluate taxonomic classification regimes (Bokulich, Kaehler, et al., 2018). Systematic evaluation of animal metabarcoding studies is growing but remain limited in scope; synthetic mock samples have been used to explore the potential for alternative primer use (Beng et al., 2016), while biological mock samples have been used to improve quality filtering of spurious sequence variants (Jusino et al., 2019) and to evaluate the utility of PCR replicates (Galan et al., 2018). In addition, few studies have used real data (i.e., actual diet samples) to offer insights into the effects of sequencing platforms (Divoll, Brown, Kinne, McCracken, & O'Keefe, 2018) or abundance filtering parameters (Alberdi et al., 2018). We build upon these analytical considerations by using real and biological mock data to illustrate how both software choice and subsequent filtering criteria impact the interpretation of community richness and composition, a common focus of diet analyses.

One of the first considerations in an amplicon study is using a denoising approach or clustering to define the representative sequences in a dataset. Denoising programs like DADA2 (Callahan et al., 2016) or Deblur (Amir et al., 2017) generate error models to address potential sequence errors, while clustering programs group sequence variants into operational taxonomic units at some user‐defined similarity (Rideout et al., 2014). These have been explored empirically in a microbial setting (Glassman & Martiny, 2018). While the observed differences were small in that study, practical reasons such as database independence and the potential to preserve sequence diversity suggest that using amplicon sequence variants (ASVs, distinct biological sequences) is more advantageous than operational taxonomic units (OTUs) (Callahan, McMurdie, & Holmes, 2017). Denoising methods have yet to see wider adoption in diet metabarcoding studies (but see Vesterinen, Puisto, Blomberg, and Lilley (2018)); most studies use clustering (Bohmann et al., 2018; Clare, Chain, Littlefair, & Cristescu, 2016; Czenze et al., 2018; Divoll et al., 2018; Kaunisto, Roslin, Sääksjärvi, & Vesterinen, 2017). We compare these denoising and clustering‐based approaches using biological mock communities and bat guano samples to highlight their effects on common diversity metric results.

As with sequence filtering considerations, assigning taxonomic information to sequence variants is fraught with decisions that can significantly impact animal diet interpretation. Classification accuracy is affected by two related issues, algorithm and reference database selection. With respect to database construction, relatively few database resources are available for conventional animal diet metabarcoding studies. In particular, the Barcode of Life Database (BOLD) (Ratnasingham & Hebert, 2007) serves as the principal resource among arthropod‐specific metabarcoding studies, while GenBank (Benson et al., 2013) is often used for nonchordate investigations. In contrast to microbial reference databases such as UNITE (Nilsson et al., 2019), Greengenes (DeSantis et al., 2006) and SILVA (Pruesse et al., 2007), BOLD and GenBank are continuously updated and lack the kind of versioned history found among microbial reference databases. Thus, two studies using the same database (e.g., BOLD) in the same discipline conducted less than a year apart may differ by tens to hundreds of thousands of reference sequences. The effects of database composition on results are unclear and make it challenging to understand if differences observed between studies are due to meaningful biology or database curation. Thus, we examined how the effects of reference database choice and subsequent filtering criteria (clustering radius) on reference composition.

Taxonomic classification of representative sequences varies considerably among animal diet metabarcoding studies but can directly affect results. For example, one may choose to classify sequences using local alignment with software like BLAST (Camacho et al., 2009) or a global aligner‐like VSEARCH (Rognes, Flouri, Nichols, Quince, & Mahé, 2016). Alternatively, kmer‐based classifiers such as SINTAX (Edgar, 2016) or Naive Bayes classification (Bokulich, Dillon, et al., 2018) can be used to taxonomically assign sequences. The BOLD API offers its own classifier although few details describing the underlying algorithm are currently available (Ratnasingham & Hebert, 2007) and unlike most other classifiers no source code is publicly documented. Additionally, hybrid methods are available wherein multiple distinct classifiers converge on a best match (Bokulich, Kaehler, et al., 2018; Palmer et al., 2018). We benchmarked taxonomic classification using both a mock community and bat guano samples to evaluate relative classifier completeness and accuracy. Our selection of classifiers was not meant to be exhaustive; rather, we chose classifiers that represent actively developed software using distinct classes of commonly used methodologies (alignment, kmer, and HMM‐based).

Finally, estimating the abundance of specific taxa in a diet, for example, proportion of the diet comprised of mosquitoes, is challenging. While creating and classifying representative sequences are processes common to most animal diet metabarcoding projects, only recently have researchers explored diversity assessments using relative abundances (RA) of sequence counts instead of transforming these counts into a presence–absence (PA) matrix of samples and observed sequence variants (reviewed by Deagle et al., 2019). Essential to the debate about the appropriateness of RA versus PA transformations is a more fundamental point: Researchers should have some insight into the presence and distribution of sequences before considering transformations. While mock communities do not represent the true complexity observed in actual diet samples, mock samples are essential for ground‐truthing bioinformatic processes such as filtering. Specifically, mock samples provide a positive control of known sequence identities and therefore enable evaluation of the frequency at which low‐abundance sequence artifacts are generated. Mock communities can provide an empirically derived filtering strategy and assess the likelihood and relative abundances of unexpected sequences (Palmer et al., 2018).

We assessed these sequence processing and classification methods using four libraries of COI data generated from an ongoing bat diet study that included a biological mock community sample and hundreds of bat guano samples for each sequencing run. While mock data provide a ground truth when evaluating different sequence filtering and classification techniques, guano data can provide relative comparisons of these procedures using the more complex samples typically found in animal metabarcoding projects. In addition, we sought to understand how our interpretations of apparent diversity within and between samples are influenced by such count transformations, and how the count data are influenced by the specific filtering program. Notably, while we tested each of these three broad processes separately, the entire workflow is interconnected. Our aim is not to present a single best pathway for all animal metabarcoding projects, but to illustrate how these processes are affected by program or parameter choices. We performed each of these analyses using QIIME 2 (Bolyen et al., 2019) to allow for increased methods transparency, reproducibility, and use of open‐source tools for diet metabarcoding.

## 2 MATERIALS AND METHODS

We evaluated sequence filtering and classification regimes using both bat guano and biological mock community sequence data. Biological mock community data provide an important means to compare how observed outcomes deviate from expected results, while bat guano samples provide a more realistic evaluation of how certain bioinformatic decisions impact an analysis.

Complete details including scripts and data files used in this manuscript are available at the GitHub repo for this project: https://github.com/devonorourke/tidybug. We created a separate document for each aspect of the project and also further describe the wet‐bench work such as sample collection, primer design, PCR conditions, quantitation of amplicons, pooling of libraries, and link the scripts used in each of the comparisons for sequence processing, database construction, and classification.

Supplementary tables and figures referred to within this document are posted to this GitHub repository at the following address: https://github.com/devonorourke/tidybug/blob/master/SupplementaryFiguresTables/

### 2.1 Mock samples

Mock community samples were constructed specifically for arthropod diet analyses of COI gene fragments (Jusino et al., 2019). DNA was extracted from vouchered arthropod specimens, and a large (~600 bp) fragment of the COI gene was amplified and cloned into a plasmid for Sanger sequencing. A smaller gene fragment (~180 bp) of each specimen was amplified using the ANML primers described in Jusino et al. (2019). These PCR products were pooled in equimolar ratios for use in our experiments. The mock community used in this experiment included 24 unique representative arthropod COI sequences derived from 23 distinct taxa; notably, two sequences are variants from the same species (*Harmonia axyridis*). Species‐level taxonomic identities were assigned by a trained entomologist's visual identification in 21 of 24 cases. For the three incompletely assigned specimens, we aligned Sanger COI sequences using NCBI BLAST, and the shorter COI sequences within that region defined by the ANML primers using the BOLD search engine. Two of the three specimens were identified in both databases as having a single representative species containing at least 90% coverage and 98.5% identity for BLAST and 100% identity for BOLD. Notably, BOLD translates sequence data into amino acid format, and thus, the exact filtering parameters are not directly comparable. The remaining unidentified specimen was not used in our classification analyses, but was retained for the section on denoising. Fasta files containing the sequence and taxonomic information for each mock sample are available on our GitHub repo https://github.com/devonorourke/tidybug/tree/master/data/mock_community

### 2.2 Guano samples

Individual fecal pellets were passively sampled weekly from sites throughout Northeastern United States (Figure  S1). Guano samples were collected using sterile forceps; pellets were collected on clean plastic sheets and stored in tubes filled with 1 ml storage buffer (3.5 M ammonium sulfate, 16.7 mM sodium citrate, 13.3 mM EDTA, pH 5.2) and then stored at −80°C. Ultimately 1,648 guano samples were used in our diversity and classification analyses. A subset of these samples specific to a single location (Fox State Forest) were used as examples for the alpha beta diversity assessments—these included 167 samples initially, and 82 samples following standard or extra filtering regardless of denoising program.

### 2.3 Laboratory work

Individual fecal pellets were extracted using DNeasy PowerSoil Kits (Qiagen). Samples were eluted with 60 µl of elution buffer, with up to eight extraction blanks per 96‐well plate. We used a dual‐indexed primer design following Kozich, Westcott, Baxter, Highlander, and Schloss (2013) to amplify a 181 bp COI gene fragment. The COI‐specific primer region of this construct is identical to that used to generate the mock community data. Four libraries were sequenced using an Illumina MiSeq platform (Illumina) using v3 chemistry with 600 cycles of 2 × 300 bp paired‐end read lengths. While each guano sample was sequenced once, the same mock community was independently amplified and pooled into every library. We describe the four sequencing runs as Libraries A–D and specify which library the mock sequences were derived.

### 2.4 Sequence processing with QIIME 2

We used the QIIME 2 v2018.11 (Bolyen et al., 2019) environment to import raw reads and trimmed with Cutadapt (Martin, 2011), retaining all paired‐end reads (not merged) with a minimum length of 175 bp for denoising or clustering. Representative sequences were identified starting with the same trimmed, unmerged input data, using one of three amplicon processing programs (hereafter termed *denoising methods*). Note that VSEARCH is not technically a denoiser, but we use the term to refer to all three programs in the general sense that they attempt to collapse the entirety of a dataset into representative sequence variants, whether they be OTUs or ASVs. The OTU clustering approach with VSEARCH mirrored the parameters outlined at the VSEARCH Wiki GitHub page (https://github.com/torognes/vsearch/wiki/VSEARCH‐pipeline), but were executed in a QIIME 2 environment, and included merging paired‐end data, dereplicating, clustering at 98% identity, de novo chimera filtering, and a final clustering of remaining sequence variants at 97% identity. For the Deblur pipeline, we altered two parameters from their default: ‘‐‐p‐min‐reads 2’ and ‘‐‐p‐min‐size 1’ ensuring that only singleton reads were discarded and all singleton ASVs were retained. Bat‐associated COI sequences were identified from filtered reads and removed, and then, representative sequence (fasta‐like) files and frequency tables of sequence counts (OTU table‐like) were merged for all libraries. See https://github.com/devonorourke/tidybug/blob/master/docs/sequence_filtering.md for complete details regarding sequence processing workflows for each pipeline.

Mock ASVs were aligned to the expected mock community references. We categorized each representative sequence by alignment scores into one of three groups: 100% identities as “exact,” 97%–99% as “partial” matches, and those <97% identity as “miss” matches. Only those amplicons with at least 97% coverage were considered in the analysis. The second dataset of bat guano consisted of the remaining ASVs identified from guano samples that were filtered for host and mock COI sequences.

Mock community and bat guano samples were then further filtered to remove low‐abundance reads using two alternative strategies. Recommendations for removing samples with overall low read abundances are described in the microbiome literature (Bokulich et al., 2013; Caporaso et al., 2011; Thompson et al., 2017). Best practices for data filtering have not been determined for animal diet metabarcoding projects despite being used with various parameters (Divoll et al., 2018; Mata et al., 2019). We applied two simple filters to the default (basic) outputs of the pipelines: First, a standard filter required (a) dropping any sequence variant observed in just one sample across the entire dataset and (b) retaining only samples with ≥5,000 total filtered reads. Second, an extra filter incorporated the standard filters, and subtracted a single, fixed integer from each element of the feature table. The integer used in the extra filter is obtained on a per‐library basis and was defined as the maximum count value observed of “miss” sequence variant in the (library‐specific) mock sample. This second filter removed sequence variants with very low read counts while scaling with library throughput, given that increasing number of artefacts are likely related to sequencing depth (Deagle et al., 2019). Notably, while guano samples may have contained as few as 5,000 reads per sample, all mock libraries contained at least 91,000 reads prior to applying the “standard” or “extra” filtering.

### 2.5 Sequence diversity analyses

We compared the mean ranks of the number of mock ASVs for each of the filtered datasets using a Kruskal–Wallis nonparametric test and compared pairwise differences using a Dunn's test. These tests identified whether denoising programs (DADA2, Deblur, and VSEARCH) produced different exact, partial, or poorly aligned (miss) ASVs; we applied these tests separately for each of the three filtering parameters (basic, standard, or extra). A subset of guano samples derived from a single site (Fox State Forest) was also assessed using the same Kruskal–Wallis test to illustrate how denoising and filtering may alter the subsequent interpretation of a single experiment. This analysis contained 14–46 samples per month, depending on denoising and filtering parameters used. For bat guano data, we tested whether the distribution of read abundances observed among ASVs differed between denoised datasets for each filtering parameter by applying a Wasserstein test. In addition, a linear model of richness (ASVs observed) for each of the three pairwise comparisons of denoising programs was generated at each filtering level.

We used Hill Numbers 0, 1, and 2 (equivalent to the diversity metrics observed richness, Shannon's Entropy, and Simpson's Index) to investigate whether denoising program and filtering platforms impacted abundance unweighted and weighted alpha diversity estimates for mock and guano samples. Samples were rarefied to 5,000 reads per sample prior to estimating alpha diversity. We applied a separate Kruskal–Wallis test to identify group differences for alpha diversity among denoising programs for each filtering regime and Hill Number. A post hoc Dunn's test was used to identify pairwise differences among denoising programs.

We tested whether variation of ASV composition within selected bat guano samples treated with a particular denoising and filtering regime was less within than between groups using a PERMANOVA. We investigated these effects using three distance measures: an incidence‐based measure (Dice‐Sorensen), and two quantitative metrics (Bray–Curtis and Morisita–Horn); datasets were rarefied to a depth of 5,000 sequences per sample. We applied these tests only among samples collected at a single location (Fox State Forest, Hillsborough NH) from April to October 2016 and included date of collection in the model.

All statistical analyses were performed in R (R Core Team, 2018) version 3.5.1 imported with the QIIME2R package (Bisanz, 2018) and processed with Tidyverse (Wickham, 2017), Reshape2 (Wickham, 2007), Phyloseq (McMurdie & Holmes, 2013), and Vegan (Oksanen et al., 2018) packages. We relied on additional R packages to create figures, including cowplot (Wilke, 2017), ggpubr (Kassambara, 2018), ggrepel (Slowikowski, 2018), ggridges (Wilke, 2018), stringi (Gagolewski, 2019), scales (Wickham, 2018), and viridis (Garnier, 2018).

### 2.6 Database curation

We compared three databases to assess the effects of sequence acquisition and curation on COI profiling results. First, the AMPTK program (Palmer et al., 2018) contains scripts to access a precompiled COI database containing both arthropod and chordate records derived exclusively from BOLD—this dataset is herein referred to as “Palmer.” Second, a dataset containing Eukaryote‐wide COI references derived exclusively from GenBank (Porter & Hajibabaei, 2018a) was downloaded using the v3.2 reference sequences and is referred to as “Porter.” We restricted our comparisons across datasets to dereplicated arthropod records, which necessitated further filtering both datasets. The Palmer sequences required removing all chordate data, while the Porter dataset required the removal all nonarthropod records, applying the least common ancestor (LCA) algorithm to the remaining arthropod records, and then dereplicating.

A third database was curated following our own methods as described below—this dataset is herein referred to as “tidybug.” We accessed arthropod records from the BOLD database using the bold R package (Chamberlain, 2017) on 24 February 2019. We then applied a custom script to retain only those records that contained the “COI‐5P” marker code and removed all records that failed to include at least family‐level taxonomic information. Prior to dereplication, we adapted methods used to format the SILVA database that incorporated an LCA approach to retain only taxonomic information where redundant sequences contain disparate classifications. Finally, to compare how clustering databases can impact the composition and completeness of information of available records, we applied the same LCA approach to cluster our curated database at 99%, 97%, and 95% identities.

### 2.7 Taxonomic classification

We first compared the accuracy of taxonomic classifiers using the mock community sequence data. We assigned an expected taxonomy to each of the 23 mock samples from class through species level and compared the proportion of true‐positive, false‐positive, and false‐negative classification assignments from each of the five classifiers tested. We quantified precision as Taxonomic Accuracy Rate (TAR) and recall as Taxonomic Discovery Rate (TDR) following the conventions used in a previous microbiome classifier benchmarking study (Bokulich, Kaehler, et al., 2018). TAR is calculated as the fraction of observed taxa that were expected at a given taxonomic level (TAR = true positive/(true positive + false positive)), while TDR represents the fraction of expected taxa observed at a particular taxonomic level (TDR = true positive/(true positive + false negative)).

We compared taxonomic classification accuracy of five different classification methods. Three of these methods are implemented in the QIIME 2 plugin q2‐feature‐classifier: alignment with BLAST (classify‐consensus‐blast) or VSEARCH (classify‐consensus‐vsearch) followed by least common ancestor consensus taxonomy assignment; and a kmer‐based Naive Bayes taxonomy classifier implemented with scikit‐learn (Pedregosa et al., 2011). We added a second kmer‐based method using the implementation of the SINTAX algorithm in VSEARCH (referred to as “vsearch‐SINTAX” below) (Rognes et al., 2016). We also classified our mock community using the BOLD API. We altered parameters for both alignment and kmer‐based classifiers: percent identities of 95%, 97%, and 99% were tested for VSEARCH + LCA (q2) and BLAST + LCA (q2) classifiers; confidence thresholds of 30%, 50%, 70%, 80%, and 90% were tested for Naive Bayes (q2) and the vsearch‐SINTAX classifier. We applied a custom R script to the BOLD API output to mirror the parameters present in BLAST and VSEARCH: first, to retain only matches with greater than either 95%, 97%, or 99% identity, and second, to apply an LCA.

We examined consensus among classifiers by calculating the number of common and distinct taxonomic names applied to bat guano ASVs. Collectively, four classifiers (VSEARCH + LCA (q2), BLAST + LCA (q2), Naive Bayes (q2), and vsearch‐SINTAX) shared the same tidybug database information. We also explored how the BOLD classification engine would compare to the other classifiers, but this comparison was limited because their classification parameters are not publicly documented, nor is the specific database used for classification defined (i.e., there is no single file to download that represents the BOLD database at the time in which their taxonomy API is queried). Our intention in comparing classifiers with actual guano data was to assess the instances in which classifiers agree or disagree with respect to a given taxonomic name at a particular level (from class through species). We applied a 97% identity threshold for the BOLD API as well as VSEARCH + LCA and BLAST + LCA q2‐classifiers and applied a 70% confidence threshold for the vsearch‐SINTAX and Naive Bayes (q2) classifiers.

Because we ultimately observed the BOLD method to generally under‐classify our guano data, we investigated the particular ASVs that BOLD failed to assign a species name, but where the non‐BOLD classifiers assigned a species name. We retained instances that satisfied the condition in which an ASV included a species name for all non‐BOLD classifiers, but was no species name was assigned for that ASV by the BOLD classifier. Among these cases, we next determined whether those species names that were assigned by non‐BOLD classifiers were present in any other ASVs classified by BOLD—this demonstrates that the issue relates to the classifier itself and is not a database composition issue. Consider the following example: all non‐BOLD classifiers assign ASV1 as species X, but BOLD fails to assign a species name to ASV1. We were interested whether or not species X was assigned by BOLD for all the remaining ASVs in the dataset. Thus, we divided the ASVs into two bins: (a) those ASVs that BOLD failed to assign a species name and failed to have any other ASV with a species named assigned by all other non‐BOLD classifiers; (b) those ASVs that BOLD failed to assign a species name, but did assign the missing species name shared by non‐BOLD classifiers in other ASVs. We considered two measures among these ASVs when evaluating their biological significance: the total number of reads that ASV generated in the entire dataset (i.e., reads summed across all samples), and the number of samples in which an ASV was detected.

## 3 RESULTS

### 3.1 Sequence diversity analyses

**FIGURE 1:**
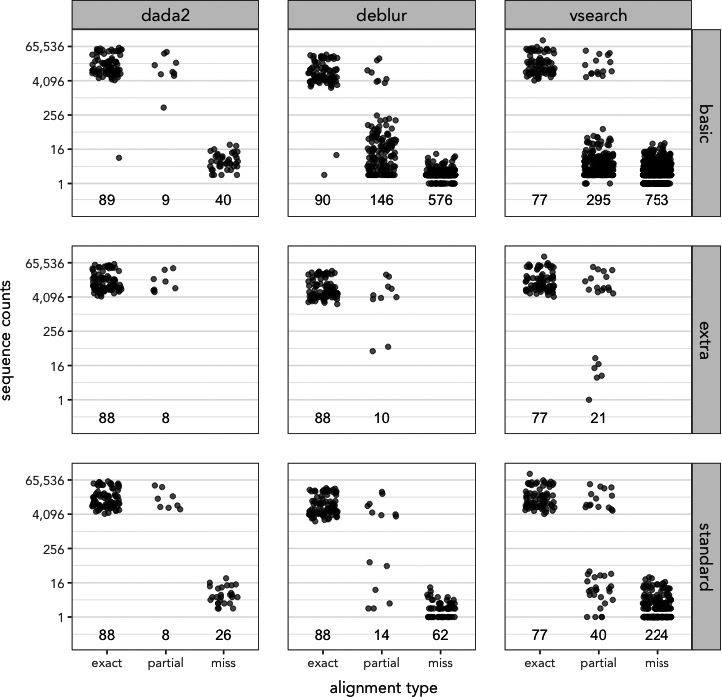
Denoising program and parameter evaluations of mock data. The number of sequences observed that are “exact,” “partial,” and “miss” matches for mock community sequences are shown for each denoising pipeline (vertical facets) and filtering parameters (horizontal facets). Values beneath each group reflect the number of ASVs combined among all four mock replicates in that group. “Exact” matches reflect 100% alignment identity between ASV detected in mock community and a known mock sequence, “partial” reflects between 97% and 99.9% identity, and “miss” represents an ASV with <97% identity to known mock sequences

The choice of both denoising program and filtering can influence the number of mock ASVs observed in dataset (Figure ). A Kruskal–Wallis test for differences in mean rank read abundances indicated significant differences between denoising groups for each filtering strategy: basic (H(2) = 270, *p* ≤ .001), standard (H(2) = 110, *p* ≤ .001), and extra (H(2) = 33, *p* ≤ .001) types. A post hoc Dunn's test with Benjamini–Hochberg correction revealed significant differences at *p* ≤ .05 for most pairwise comparisons: DADA2 and Deblur for basic, standard, and extra‐filtered datasets; DADA2 and VSEARCH for basic and standard filtered data, but not for extra; Deblur and VSEARCH for standard and extra‐filtered data, but not for basic. The majority of differences among observed ASVs between these denoising programs and filtering parameters were driven by the presence or absence of unexpected ASVs: either those partial sequences containing either 99%–97% sequence alignment to a known mock reference, or the miss ASVs containing less than 97% similarity to any expected mock sequence (Figure 1). DADA2 contains fewer partial ASVs (9) than Deblur (146) or VSEARCH (295) for basic‐filtered data, and fewer miss ASVs (40) than Deblur (576) or VSEARCH (753). The number of unexpected mock ASVs is reduced by applying either standard and extra filtering parameters, and however, given that the mock DADA2‐denoised dataset had so few unexpected ASVs to start, these additional filtering strategies appear particularly valuable for pipelines incorporating either Deblur or VSEARCH.

**FIGURE 2:**
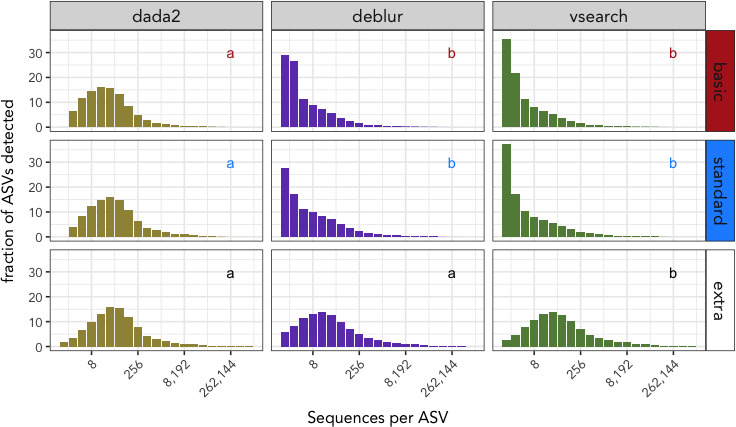
Read abundances per sequence variant among all bat guano samples. The frequency with which ASVs are detected are binned as a power of two (i.e., first bin contains singletons, second bin contains ASVs with 2–3 sequences, third bin contains 4–7 sequences). Distributions of ASV abundances are partitioned by denoising program (vertical facets) and filtering regime (horizontal facets). Letters inset for each subplot reflect statistically significant differences between each denoising group. Note that while per‐sample singleton sequences are initially discarded during raw data processing, singleton sequences can arise in the extra‐filtered dataset because a fixed number of reads was subtracted from every ASV on a per‐sample basis

Bat guano samples consisted predominantly of ASVs with low sequence abundances, yet the particular denoising method and filtering parameters affected the distribution of sequence abundances (Figure 2). The basic‐filtered DADA2 distribution was significantly different than Deblur (*W* = 752.07, *p* ≤ .001) and VSEARCH (*W* = 734.77, *p* ≤ .001), but Deblur did not differ from VSEARCH (*W* = 49.05, *p* = .754). The same pattern was observed with the standard filtered DADA2 distribution varying significantly from both Deblur (*W* = 911.61, *p* ≤ .001) and VSEARCH (*W* = 818.87, *p* ≤ .001), but not between Deblur and VSEARCH (*W* = 152.74, *p* = .342). In the case of extra‐filtered distributions, VSEARCH significantly differed from DADA2 (*W* = 877.06, *p* ≤ .001) and Deblur (*W* = 1,040.30, *p* ≤ .001), but not between DADA2 and Deblur (*W* = 316.05, *p* = .488). These differences in distributions between DADA2 and the alternative denoisers are attributed to the presence or absence of singleton and doubleton ASVs for basic or standard filtered datasets. For example, more than half of the ASVs detected among bat guano samples processed with Deblur (55.7%) or VSEARCH's (57.1%) basic output contained no more than 2 reads per ASV, while these doubleton and singleton ASVs represented just 6.5% of all DADA2 basic detections. The proportion of singleton and doubleton ASVs is reduced for all extra‐filtered datasets, but the reduction in these lowest‐abundance ASVs is greater for both VSEARCH (7.19%) and Deblur (14.4%) than DADA2 (5%).

With only 4 mock samples sequenced, we lacked statistical power to evaluate if alpha diversity estimates varied among denoising methods for each filtering regime. Nevertheless, we provide diversity estimates (Table S2) for each mock sample depending on these denoising and filtering treatments for Hill Numbers 0, 1, and 2 to illustrate that nearly all estimates for all treatment combinations fell within 2–3 species equivalents of the expected mock membership. Because the expected mock ASVs routinely generated 100‐fold greater sequence abundances compared to any unexpected ASV in a mock sample, diversity estimates incorporating abundance information had little impact.

Denoising program and filtering regime influenced the alpha diversity estimates observed for bat guano data whether or not read abundance information was incorporated (Figure S2). With the exception of Hill Number 0 (equivalent to observed richness) for basic‐filtered data (H(2) = 1.823, *p* = .402), all other diversity estimates contained significant differences among filtering parameter and denoising software groups (Table S3). A Dunn's test comparing diversity estimates was performed for each pairwise combination of denoising method and filtering parameter per Hill Number (Tables S4). These same patterns were observed when fitting a linear model to the number of ASVs observed in a given sample, depending on the denoising and filtering methods applied (Figure S3). While the correlations of ASV richness were weakest among the basic or standard filtered data, samples clustered with VSEARCH repeatedly contained 2–3x more ASVs per sample than the same samples denoised by either Deblur or DADA2. Deblur and VSEARCH often contained very similar ASV richness (*r* ^2^ = .91), after applying the extra filter, yet the correlation was lower when comparing DADA2 to either Deblur (*r* ^2^ = .71) or VSEARCH (*r* ^2^ =  .69).

**FIGURE 3:**
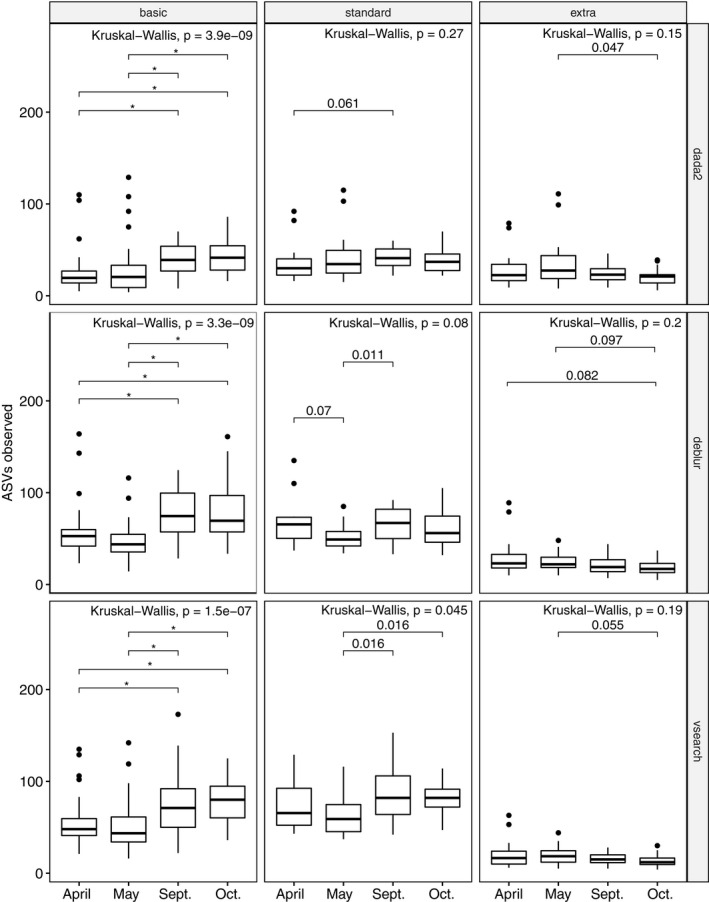
Biological meaning can change depending on the particular filtering (vertical facets) and denoising (horizontal facets) methods applied to a dataset. This analysis examined mean rank ASV richness among guano samples collected from a single site (Fox State Forest, Hillsboro, NH, USA) over four sampling months. Filtering greatly reduces the number of significantly different sampling months, whereas denoising platform can identify different significant pairwise comparisons depending on the particular filtering regime used, as well as the confidence level used to determine significance. Exact *p*‐values indicated for significant pairwise comparisons Asterisks indicate significance threshold *p* <  0.001; exact significance values indicated when 0.001 < *p* < 0.1

As one example illustrating whether filtering or denoising practices would change our subsequent interpretation of biological effect in a study, we compared the mean rank in richness per collection month for a single, well‐sampled site (Figure 3). All denoising programs identified similar differences in the mean rank of ASV richness for basic‐filtered data (H(3), *p* < .001), yet standard and extra‐filtered data varied with respect to within‐group differences depending on the denoising program used. Both the particular months and the number of significant pairwise differences between months varied depending on the filtering and denoising platform used.

We tested whether variation of ASV composition within bat guano samples from a single collection site treated with a particular denoising and filtering regime was less within than between groups using a PERMANOVA. No significant effects of denoising method or filtering parameters among either abundance‐weighted distance estimate (Bray–Curtis and Morisita–Horn) were found, although the month in which guano samples were collected was significant for both metrics (Table S5). There were no significant group interactions among abundance‐weighted distance estimates. For unweighted estimate using a Dice–Sorensen distance metric, we identified significant effects of collection month and for filtering regime, but not for denoising method, nor any interaction term (Table S5).

### 3.2 Database construction

We compared how the composition of arthropod COI records varied among three databases: two created from BOLD records (Palmer and tidybug) and one from GenBank records (Porter). While the Porter (1,280,577 total COI records; 515,780 arthropod‐specific COI records) and Palmer databases (1,617,885 total COI records; 1,565,831 arthropod COI records) contained arthropod as well as nonarthropod COI records, the tidybug database was constructed exclusively with arthropod COI records yet contained the largest number of distinct sequences overall (1,841,956 arthropod COI records). These differences in total arthropod COI references reflect a series of decisions by the researchers constructing these databases including both the source with which sequences were obtained (GenBank or BOLD), and how databases were curated (i.e., requiring complete taxonomic names for all references (or not), clustering references (or not), and the time with which records were obtained).

**FIGURE 4:**
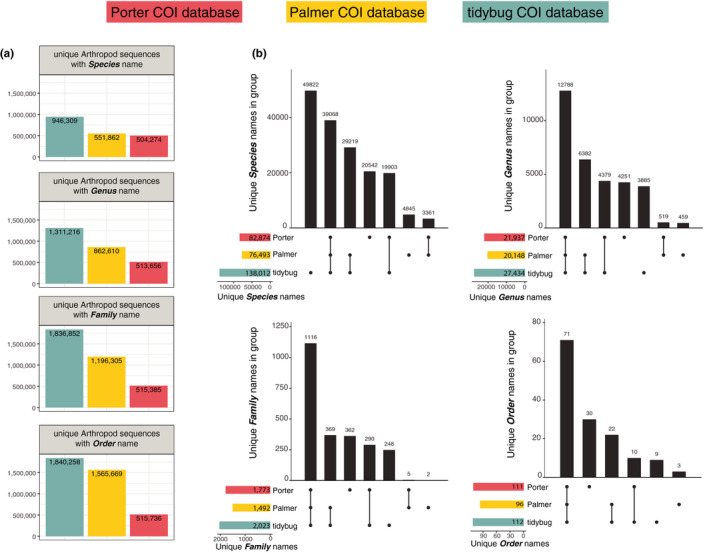
Porter, Palmer, and tidybug database comparisons. (a) Counts of dereplicated reference sequences containing complete taxonomic descriptions including the species name (top panel); lack only species name but include all others up to and including genus (second from top); lack species and genus but include family (third from top); or lack only up to order‐level names (bottom panel). (b) Counts of unique taxonomic names present among distinct and overlapping sets of databases at species, genus, family, and order levels. Total number of unique taxa present at each level for each database indicated in bars at bottom left of each panel. Number of taxa names present in a given group indicated by vertical bars with dots underneath each bar indicating group membership

The quality of a database is not only a function of how many records it contains, but also by how complete the taxonomies are for the references. For instance, references may or may not contain a name at the family, genus, or species rank. With respect to the number of unique taxa and unique sequences, the tidybug database contained more distinct records from species through order levels (Figure 4a). This difference was most pronounced between the Porter database and the Palmer or tidybug records and reflects the fact that the Porter database required all references to contain complete taxonomic names (i.e., including species rank), while neither the Palmer nor tidybug database was created with such a stipulation. Nevertheless, the tidybug database including species rank contained over 400,000 more records than the Porter database, illustrating that both filtering criteria and reference sources are important factors when constructing a database.

There are considerable differences among the number of shared taxa (found in two or more databases) and unique taxa (found in only one database) (Figure 4b). While all three databases shared many species records in common (39,068), it was in fact the tidybug database that contained the most species records (49,822) among all sets. The Porter dataset (20,542 species records) and Palmer dataset (4,845 species records) contained fewer distinct records. Order, family, and genus names were shared most frequently among all three databases, though we observed that individual databases routinely contained sizeable fractions of unique taxa. For example, while 12,788 genera were shared among all three databases, 4,251 unique genera were observed only in the Porter dataset, and 3,885 genera were reported only in the tidybug dataset. The same effect was observed among family names, with 1,116 shared among all three datasets, while 362 family names were identified uniquely in the Porter dataset and 248 found uniquely in the tidybug database.

Database composition can be further impacted by choice of reference clustering. We clustered our tidybug database at three levels (99%, 97%, and 95%) to understand the effects of clustering on taxonomic composition (Table 1). Clustering at 99% reduced the original number of dereplicated sequences from 1,841,946 to 407,356. Further clustering at 97% (265,885 records) and 95% (215,055 records) results in additional reductions in the number of representative sequences. In addition, we observed that the overall proportion of ambiguous genus and species names increases as clustering radii decrease. For example, while 5.6% of species names were ambiguous among the dereplicated dataset, 7.2% of species names were ambiguous for 99% clustered data, 11.4% were ambiguous for 97% clustered data, and 15% of species names were ambiguous for 95% clustered data (Table 1).

**TABLE 1:** Clustering tidybug database increases taxonomic ambiguity of reference names and reduces the total number of distinct sequences

Clustering percent	Level	Unique reference sequences		
		Present	Ambiguous	Missing
100%	Order	1,840,258 (99.9%)	1,688 (0.01%)	0
	Family	1,836,852 (99.7%)	5,094 (0.03%)	0
	Genus	1,311,216 (71.2%)	46,044 (2.5%)	484,686 (26.3%)
	Species	946,309 (51.4%)	103,150 (5.6%)	792,487 (43.0%)
99%	Order	406,935 (99.9%)	421 (0.01%)	0
	Family	405,906 (99.6%)	1,450 (0.04%)	0
	Genus	291,579 (71.6%)	10,734 (2.6%)	105,043 (25.8%)
	Species	230,430 (56.6%)	29,517 (7.2%)	147,409 (36.2%)
97%	Order	265,571 (99.9%)	314 (0.01%)	0
	Family	264,458 (99.5%)	1,427 (0.05%)	0
	Genus	183,905 (69.2%)	10,873 (4.1%)	71,107 (26.7%)
	Species	141,186 (53.1%)	30,339 (11.4%)	94,360 (35.5%)
95%	Order	214,737 (99.9%)	318 (0.01%)	0
	Family	213,176 (99.1%)	1,879 (0.09%)	0
	Genus	143,075 (66.5%)	12,195 (5.7%)	59,785 (27.8%)
	Species	106,370 (49.5%)	32,227 (15.0%)	76,458 (35.6%)

Note

The tidybug database was dereplicated (100%) or further clustered at one of three radii (99%, 97%, 95%). The resulting arthropod COI sequences either contained taxonomic information (Present) or lacked information (Missing) at a particular taxonomic rank (class through species). Ambiguous taxa information is created when identical sequences have distinct taxonomic descriptions (evaluated at each taxonomic rank separately). The total number of references shown for Present, Ambiguous, and Missing names are indicated and the relative percent of names contained at each clustering percent.

**FIGURE 5:**
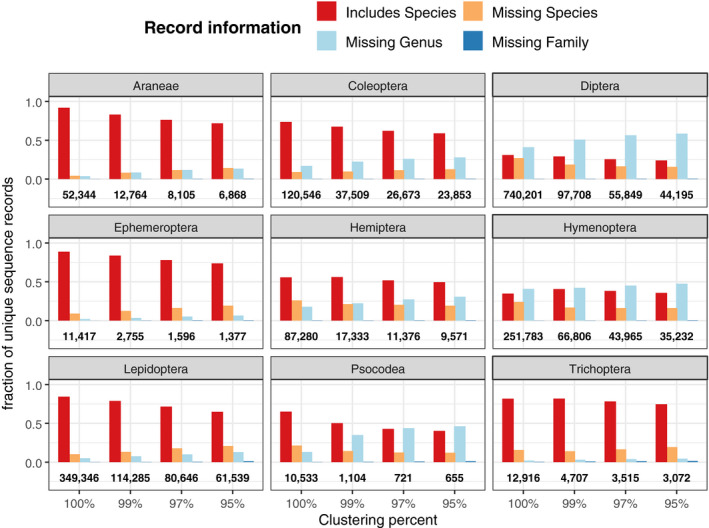
Clustering tidybug database nonuniformly reduces sequence and taxonomic diversity among selected arthropod orders. References may contain all taxonomic information (red), while other records are missing species name (orange), species and genus names (light blue), or species, genus, and family information. The fraction of sequences with various taxonomic completeness are shown for each dataset as a result of dereplication (100%) or further clustering (99%, 97%, or 95%). Total references for each order are shown below each group

We further examined whether clustering reduced sequence diversity and taxonomic ambiguity equally among nine arthropod orders containing some of the largest number of reference sequences (Figure 5). While clustering the entire dataset at 99% reduced the number of unique sequences to about 22.1% of its original size, this reduction varied among arthropod orders. The least overall reduction of references was observed among Trichoptera (36.4% of original size), Coleoptera (31.1%), and Lepidoptera (32.7%), while Diptera (13.2%) and Psocodea (10.5%) were among the orders that had the greatest reduction of sequence diversity due to clustering. In addition, some arthropod orders contained many dereplicated references that lacked either species or genus names: Diptera (41.1%) and Hymenoptera (40.9%) were the two orders containing the greatest number and frequency of such incomplete references. However, clustering at 97% resulted in a greater increase of references lacking species or genus names for Diptera (56.5%) than Hymenoptera (45.2%) (Figure 5, light blue bars).

### 3.3 Taxonomic classification comparisons

**FIGURE 6:**
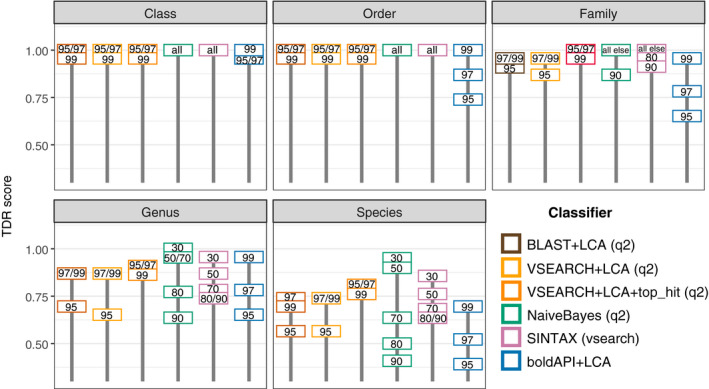
Classifier performance of mock community data as measured by Taxon Discovery Rate (TDR)—higher TDR values indicate a greater fraction of observed taxa that were expected at a particular taxonomic level. Values inside each box indicate the parameter tested for that classifier, with BLAST, VSEARCH, and BOLD values reflecting alignment percent identity, while Naive Bayes and SINTAX values indicate confidence thresholds. Values marked as “all” indicate all possible parameters within classifier tested (30/50/70/80/90 confidence thresholds), while “all else” indicate all other remaining values not specified as integers within the group. The addition of a second parameter that retains only the highest alignment score distinguishes the VSEARCH+LCA and "VSEARCH+LCA+top_hit" classifiers

All taxonomic classifiers were robust with respect to avoiding false positives: For all classifiers and all parameters, at all taxonomic levels, none of the 23 mock sequences were assigned an incorrect name. Thus, TAR scores (the fraction of observed taxa that were expected (Bokulich et al., 2013) were equal to 1 for every classifier and parameter tested at each level of taxonomic classification (phylum to species). Note that the TAR metric is not influenced by false negatives, and hence, the perfect scores indicate that none of these methods overclassified or misclassified. Using the TDR metric (the fraction of expected taxa that were observed (Bokulich et al., 2013)), differences occurred based on the classifiers and parameter settings that were used (Figure 6). For instance, the boldAPI + LCA classifier parameterized with a 95% identity threshold recorded the greatest degree of underclassification at the order, family, genus, and species levels, yet produced similar or better TDR scores compared to most other classifiers when parameterized with a 99% identity threshold. While alignment‐based classifiers BLAST + LCA (q2) and VSEARCH + LCA (q2) produced similar TDR scores for the range percent identity values tested, kmer‐based classifiers SINTAX (vsearch) and Naive Bayes (q2) exhibited a greater sensitivity to changes in the confidence parameter; specifically, lower confidence settings increased classification accuracy. The highest species‐level accuracy was observed using the q2‐feature‐classifier Naive Bayes classifier with confidence = 0.3 (Figure 6).

**FIGURE 7:**
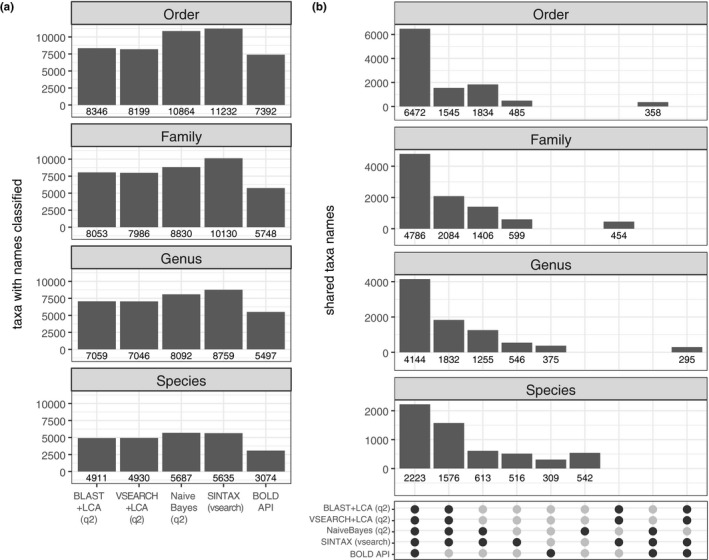
(a) Number of bat guano ASVs assigned taxonomic information at Order through Species rank for each classifier. A total of 13,407 ASVs were included in the guano dataset for potential classification. (b) Taxonomic names shared among classifiers for guano data. Vertical bars represent the number of taxonomic names matching to a given set of classifiers; each subpanel represents the taxonomic level a name is evaluated at. The particular classifiers included in a set is indicated by the shaded dots in the bottom panel. For instance, the leftmost set (all five shaded dots shaded) represents instances in which all five classifiers agree for a particular taxonomic name. The second (from left) vertical set has all except the “bold” classifier shaded, indicating the number of instances in which the four nonbold classifiers agreed on a taxonomic name that bold either lacked. Highlighted groups represent instances where set values were at least 3% of all unique taxonomic names, thus all other sets possible but not shown contained relatively few overlaps

Classifier performance was also compared on a relative basis using bat guano data: First, the frequency of ASV assignment to taxonomic name from class through species levels, and second, the degree with which one or more classifiers assigned the same taxonomic name to that ASV. More ASVs were assigned taxonomic names among order, family, and genus ranks by kmer‐based classifiers than either of the alignment‐based classifiers or the BOLD classifier (Figure 7a). While the relative proportion of ASVs assigned species names is reduced relative to more inclusive taxonomic ranks (e.x. order, family, or genus), the Naive Bayes classifier assigned the most species names among all classifiers. The BOLD classifier reported the lowest number of named taxa at each taxonomic rank. Because their API uses a closed source reference database and classification parameters are undefined, it is unclear exactly what proportion of these missing assignments are a function of the classifier algorithm itself or the database queried.

We further investigated a particular difference among classifiers: Those ASVs that failed to be assigned a species name by BOLD, but were nevertheless assigned a species name by all non‐BOLD classifiers. Among these 1,641 particular ASVs, we found that the unassigned species name (missing in BOLD, present in all non‐BOLD classifiers) is often present in alternative BOLD ASVs (Figure S4). In particular, 131 distinct species names assigned by non‐BOLD classifiers but not assigned a BOLD ASV were present in an alternate ASV classified by BOLD. Nevertheless, a larger majority, 301 species in all, were completely absent from the BOLD classifier data that were otherwise classified by non‐BOLD programs. In addition, we discovered several instances wherein tens or hundreds of unclassified BOLD ASVs likely represented just a single species. The most extreme example occurred among the Megaloptera, where we identified 283 ASVs in our bat guano samples that were not assigned a species name by BOLD but were assigned a species name by all non‐BOLD classifiers. Those 283 ASVs represented just 4 distinct species: *Chauliodes pectinicornis* (249 ASVs), *C. rastricornis* (20 ASVs), *Sialis vagans* (1 ASV), and *S. velata* (13 ASVs).

We next counted the number of instances in which classifiers assigned a similar taxonomic name to each ASV (or disagreed or failed to assign an ASV) among the 31 possible “sets” (similar to a 5‐way Venn diagram) for each taxonomic rank from order through species (Figure 7b). The largest proportion of ASVs with common names occurred within the set that included all five classifiers: The majority of taxonomic order (6,472 ASVs with common names), family (4,786), genus (4,144), and species (2,223) names were similar across all classifiers tested. The next largest set of common names existed among all non‐BOLD classifiers at family (2,084), genus (1,832), and species (1,576) levels; the kmer‐based classifiers contained the next largest set of common ordinal names (1,834). The proportion of shared non‐BOLD names increased relative to the set including all five classifiers as taxonomic levels became more exclusive: Non‐BOLD shared names were 23.8% as large as the set including all classifiers at the order level (i.e., 1,545 compared to 6,472 ordinal names), yet the non‐BOLD set was 70.9% as large as the set including all five classifiers at the species level (i.e., 1,576 compared to 2,223 species names). Thus, while fewer ASVs are assigned names at the species level than at the order level, a larger fraction of these names are not included by the BOLD classifier at the species rank than order rank. We observed that both kmer‐based classifiers agreed on many ASVs named at the order (1,834), family (1,406), and genus (1,255) ranks that were left unassigned by alignment‐based classifiers or the BOLD classifier. With the exception of ASVs with species names were shared between BLAST and VSEARCH that differed from an alternative name from at least one other classifier (1.5%), all other instances of these nonambiguous names represented <1% of available overlaps at every taxonomic level except species rank, with most instances occurring with <0.1% of the total ASV names in that set. In other words, it is exceptionally rare for at least two classifiers to assign a distinct name that differs from another classifier's alternative name.

## 4 DISCUSSION

### 4.1 Sequence filtering and diversity estimates

We sought to better understand how several common bioinformatic decisions involving sequence processing and database construction affect interpretations of diversity and subsequent classification of ASVs. Indeed, even in an ideal molecular workflow, the computational parameters invoked can potentially alter the number of sequence variants observed depending on the denoising method, and even more strikingly, completely ignore particular taxa depending on the classification strategy or database.

The first major bioinformatic decision that has yet to reach consensus in animal diet metabarcoding studies relates to identifying unique sequence variants. As has been shown for microbial studies (for which these methods were developed), denoising methods appear advantageous over OTU clustering both for retaining expected sequence variants and reducing spurious sequence variants. With respect to recalling the expected sequence variants among our mock community data, both DADA2 and Deblur worked similarly well, each detecting all 24 expected mock sequences, while VSEARCH failed to detect 1–4 expected mock members among the four sequencing runs (Table S6). The missing “exact” matches using VSEARCH highlights a problem inherent with a clustering approach to denoising: loss of information due to shared sequence similarity. Two of our mock sequences were variants of the same species; one each of the exact variants clustered together in the VSEARCH library. If these mock samples had been representative of an actual diet analysis, we would have been successful in identifying the species, and however, we would have failed to detect the intraspecific differences of distinct ASVs within the species.

Relatively few animal diet metabarcoding studies incorporate read abundance information in their diversity estimates because of uncertainty in associating biomass with sequence counts (Alberdi et al., 2018; Deagle et al., 2019). This is particularly meaningful given that our observed differences among mock samples processed by different denoising methods with basic and standard filtering is entirely attributed to low‐abundance sequences. Thus, the choice of denoising method and filtering parameters can dramatically change richness estimates for researchers using a presence–absence framework. For instance, 539 of the 604 unexpected ASVs contained 10 or fewer total sequences per ASV in just a single mock sample (libD) when processed with basic filtering using VSEARCH. Deblur follows a similar pattern, retaining many sequence variants with extremely low abundance: the same basic filter dataset from that same sample contains 525 total variants, yet all 364 “miss” variants contain 11 or fewer sequences per ASV. Yet only after applying the extra filter were all unexpected sequences removed from each sample, and every denoising method required had at least some unexpected ASVs removed by this filter. Discarding observed sequence variants with low abundances on a per‐sample basis or on a per variant basis operates with the assumption that rarity is more indicative of sequencing error than true biological variation. For experiments that do not include positive controls with their true samples, one is left with the challenge of arbitrarily assigning what minimum read count to use. Our mock data suggest that DADA2 is most effective at eliminating low‐abundance errors and would be particularly useful in situations whereby no additional filtering threshold can be empirically derived.

For researchers particularly interested in evaluating richness only, as is often the case in COI diet work, our example guano dataset suggests that simply removing low‐abundance data are not sufficient to resolve observed differences in richness between denoising platforms (Figure 3 and Figure S2). While VSEARCH and Deblur are highly similar with respect to their per‐sample ASV richness estimates when an extra filter was applied, both VSEARCH and Deblur are much more variable with respect to per‐sample ASV richness relative to DADA2, though not unidirectionally greater or lower. We performed one small test with bat guano collected across 4 months at a single site and found that indeed our interpretation of biological patterns could arise simply from the bioinformatic choices we made with respect to denoising platform and filtering (Figure 3). We observed more differences between monthly ASV richness in these select bat guano data than the filtered data with low‐abundance reads removed. Yet even after applying these filters, the significantly different Months varied among denoising platforms. Despite our findings that DADA2 are least affected by retaining spurious, low‐abundance sequence variants, these comparisons highlight that a richness estimate by any of the proposed denoising approaches are particularly sensitive to low‐abundance variants. While these may be of great interest in a microbial setting, they are less likely to be important factors in bat diets, and thus, if researchers are adamant in using a clustering approach over a denoising technique, modest filtering of low‐abundance reads is advised.

Because of a dependence on transforming read abundances to presence–absence, diversity estimates in animal metabarcoding studies have historically been limited to richness for intersample similarity and one of a few indices (e.g., Dice–Sorensen or Raup–Crick) for intrasample analyses. Incidence‐based approaches are often justified as a more appropriate choice compared to using relative abundances of sequences due to the challenges of associating counts to biomass (Clare, 2014; Deagle et al., 2019). Yet as Deagle et al. (2019) observe, “to accept the notion that relative sequence counts provide no meaningful information would mean that, within one sample, a few DNA sequences from one food taxon are equivalent to 10,000 sequences from another.” Sequence diversity estimates among bat guano samples were sensitive to both denoising method and filtering parameters whether or not abundance information was used to estimate alpha diversity (Figure S2). However, it is possible that differences between denoising platforms and clustering are reduced once the data are classified. For instance, the additional ASVs observed in DADA2 may be a result of intraspecific variation. Indeed, this was observed in our mock community with *H. axyridis* (where two separate vouchered specimens of the same species had different COI sequences). The extent with which this phenomenon is observed will vary among experiments. We suggest that a practical approach would include first denoising with a program like DADA2 that retains as much accurate sequence variation as possible, then let the researcher determine whether or not downstream analyses warrant further clustering using either sequence or taxonomic information.

In contrast, estimates of beta diversity for bat guano data were robust to the denoising software of filtering parameters when read abundances were incorporated (Table S5). Similar findings have been reported for microbial studies (Bokulich et al., 2013). However, filtering, but not denoising software, was a significant factor for the unweighted Dice‐Sorensen model. Our mock community suggested that a clustering approach generates many more low‐abundance false‐positive sequence variants, and thus, it appears that filtering out most low‐abundance reads was sufficient to observe community composition variability in our dataset using actual bat diet samples.

Somewhat counterintuitively, conventional COI community composition analyses often reject the notion of including read abundances to avoid bias; we find that the particular filtering parameters the researchers invoke can itself a potential source of bias. Notably, our work is not intended to determine whether using read abundances is appropriate for a particular animal diet study, but simply to highlight that detections and relative abundances in diversity estimates remain sensitive to the denoising programs and filtering criteria applied.

### 4.2 Database construction

Assigning taxonomic information to a set of sequence variants requires a reference database, yet there is no single curated and versioned resource widely used in animal diet studies, making comparisons among studies extremely difficult. While BOLD had previously presented packaged versioned releases, this practice ended in 2015. Motivated by the fact that these same complications persist for users relying on NCBI resources, Porter and Hajibabaei (2018b) created a pipeline that makes versioned releases manageable. However, their database construction choices may reflect the needs of their experiments but not for others. For instance, their requirement that all records contain species‐rank names prioritizes taxonomic information over sequence diversity and a more diverse database containing records lacking species or genus‐rank names may be preferred. As shown in Figure 4b, the two major resources of arthropod COI records may be viewed as complementary. While no single database will be sufficient for all animal metabarcoding projects, versioned resources are essential for ensuring that unique properties between experiments reflect differences in biology and not the reference databases. The recent beta release of RESCRIPt (Bokulich, Robeson, & Dillon, 2020) is one such tool that may bridge the gap in designing versioned databases curated specific to COI diet analyses.

We hope future versioned databases will explore the construction criteria we examined: filtering references with taxonomic ambiguity, dereplicating sequences, and clustering related sequences. Porter database consists nearly exclusively of full taxonomic identities—references that include species‐rank names. The Porter dataset was constructed initially to contain only records with named species, and however, by failing to dereplicate their records, there are over ten thousand instances where identical sequences contain distinct taxonomic identities. In this case, the LCA algorithm reduces these records to a common shared taxonomic level, eliminating species‐level information and highlights that dereplicating can reduce the total number of available records when a consensus LCA process is applied. For example, our “original” BOLD arthropod records contained over 3.1 million sequences, while just 1.8 million of these references remained in our dereplicated dataset. Dereplication is essential to database construction, and we opted for a consensus LCA approach instead of a majority method to avoid potentially over‐classifying the reference records.

Clustering reference sequences is performed to alleviate the computational burden of alignments, phylogeny‐building, and classification by reducing the number of highly similar reference sequences. Yet a tradeoff occurs between computational burden and compositional representation: Clustering makes searching a database faster, but the number of potentially distinct sequences and taxonomic identities are fewer. An additional problem with clustering arises from the fact that groups of taxa may have distinct evolutionary rates and thus are differentially impacted by applying a single value when related sequences are merged. For example, there is greater variation in COI sequence in Coleoptera than Lepidoptera (Pentinsaari, Salmela, Mutanen, & Roslin, 2016). Clustering reduced the number of the most abundant arthropod orders in a nonuniform manner. For example, while there are twice as many unique Diptera sequences in the tidybug database as Lepidoptera, yet clustering at 99% identity resulted in fewer overall dipteran sequences than lepidopteran. If clustering is a necessity, the dynamic clustering approach used by UNITE may be preferred to the fixed binning approach currently, but it remains unresolved exactly what clustering radii are appropriate for each taxonomic level.

### 4.3 Taxonomic classification

Assigning taxonomic identities to sequences is a primary goal in animal diet studies but classification method and parameter choice can alter biological inference. Other fundamental decisions on how taxa are filtered are equally important though often less well documented such as applying an LCA process (Galan et al., 2018). Some researchers filter acceptable taxa outside of expected geographic boundaries (Divoll et al., 2018; Vesterinen et al., 2016), although this decision may preclude many undiscovered taxa. Moreover, once a classifier is chosen, optimizing parameters for an experiment requires ground‐truthing, yet to our knowledge comparing various classifiers and parameters using biological samples with known taxonomic identities exists only for microbial amplicon data (Almeida, Mitchell, Tarkowska, & Finn, 2018; Bokulich, Kaehler, et al., 2018) and microbial (Gardner et al., 2019) and viral (Sczyrba et al., 2017) metagenomes. Among alignment‐based classifiers, reducing the percent identity resulted in more under classified sequences unless a “top‐hits” parameter was invoked (Figure 6). When a 95% or 97% identity threshold is used, multiple candidates with distinct taxonomic names occurred; because we apply an LCA process to these candidates where the resulting taxonomic name must be unique, generating a more inclusive group of matches reduces the likelihood that a single species name (or genus at 95% identity) is reported. However, invoking the “top‐hit” parameter retains only the highest alignment score, thus the LCA process is only applied if multiple best scores are reported.

In evaluating the distinctions among parameter settings within a classifier, we also discovered that the reported under classification was likely inflated because of incomplete reference names persisting among the top‐hits of a search alongside an expected (and complete) reference name. For instance, when classifying with VSEARCH + LCA, three different mock community members (IM39, IM42, and IM44) each contained the expected species name and a reference with at least as good an alignment score but a name that was either “Ambiguous” or lacked any information at the species and/or genus rank. In each of the three cases, an investigation of the BOLD BIN associated with the ambiguous reference pointed to a group containing just one named species—the expected name. This problem highlights the interconnectedness between bioinformatic processes: A more refined database curation step that removes these ambiguous references will reduce the instances in which sequences are under classified.

Kmer‐based classifiers offer an orthogonal method to classify samples without demanding a fixed percent identity. Kmer‐based classifiers nearly always assigned order names to our bat guano data, while alignment‐based approaches often returned equivalent ASVs as undefined (Figure 7a). Lowering the percent identity threshold from our conservative value of 0.97 to something less would undoubtedly retain more of these undefined taxa. However, because we apply an LCA process to all hits retained, an inherent tradeoff exists between precision and recall. For an alignment approach, lowering the percent identity may result in fewer undefined ASVs (increasing recall), but the proportion of ASVs with species‐level information will also be reduced (decreasing precision). Thus, kmer‐based approaches offer an alternative to this problem in situations in which far less of a full sequence is needed to assign some degree of taxonomic identity.

Collectively, these results suggest that the COI marker is an excellent candidate for classification optimization, though given the breadth of possible COI targets investigated among animal diet metabarcoding experiments additional mock datasets are necessary to better understand how such optimizations may vary across phyla. We encourage researchers to assign taxonomy to their datasets using a range of classifiers. Indeed, among non‐BOLD classifiers, a kmer‐based approach can be especially useful at retaining class and order information that alignment‐based classifiers would otherwise discard, but caution that preprocessing of training data for amplicon length, quality, and chimera filtering are important and necessary when using a kmer approach. Furthermore, the reduction in named taxa for VSEARCH or BLAST can be explained by the fact that these alignment‐based classifiers apply an LCA process after identifying potential matches while kmer‐based approaches do not. The consensus threshold used for assigning taxonomy is adjustable and may require further optimization for COI sequence classification. As with our database construction investigations, there are many parameters to consider when classifying animal metabarcoding data. Our work provides a template for further investigations, and the community of animal diet researchers would be well served to create a more robust and phylogenetically diverse mock dataset to identify best practices for classification.

## 5 CONCLUSIONS

Our work highlights the need to perform comparisons among the frequently adopted bioinformatic tools used in animal diet metabarcoding studies. Many of these tools are capable of producing different results depending on the pipeline invoked. Mock community results suggest that DADA2 is a superior tool at ensuring the expected community is best represented while reducing ASVs that are not expected. These same sensitivities apply to alpha diversity estimates that incorporate read abundances, although we failed to detect such a pattern among various beta diversity metrics in our bat guano dataset.

Database composition varied depending upon the reference source, whether taxonomic completeness requirement filters were applied, if an LCA was used, and whether references were clustered. Clustering reduced taxonomic diversity and increased the proportion of ambiguous names, particularly at the species and genus ranks. Further, the reduction of sequence content and taxonomic information is not reduced uniformly among many arthropod orders. We encourage researchers to consider combining reference databases when curating their own set of sequence and taxonomic records given our discoveries of the number of unique taxonomic records available among disparate database repositories and making versioned releases of these datasets.

A larger fraction of bat guano ASVs were assigned order, family, and genus names among kmer‐based classifiers, suggesting that classifier choice can alter the subsequent interpretations of community composition in a dataset. More expansive and phylogenetically diverse mock communities and in silico tests will be useful for identifying which classifiers are best served to balance the desire to classify as many taxa at as many levels as possible without introducing false indications of certainty where taxonomic ambiguity is more appropriate.

## CONFLICT OF INTEREST

The authors declare no financial interest in the opinions or materials presented in this manuscript.

## AUTHOR CONTRIBUTION

**Devon O'Rourke:** Conceptualization (lead); Data curation (lead); Formal analysis (lead); Investigation (lead); Methodology (lead); Project administration (lead); Visualization (lead); Writing‐original draft (lead); Writing‐review & editing (lead). **Nicholas Bokulich:** Methodology (supporting); Visualization (supporting); Writing‐original draft (supporting); Writing‐review & editing (equal). **Michelle A. Jusino:** Resources (supporting). **Matthew D MacManes:** Resources (equal); Writing‐review & editing (equal). **Jeffrey Foster:** Conceptualization (supporting); Formal analysis (supporting); Funding acquisition (lead); Methodology (supporting); Resources (lead); Visualization (supporting); Writing‐original draft (supporting); Writing‐review & editing (equal).

## Supporting information

Supplementary Material

## Data Availability

Documentation and scripts are available at the project's GitHub repo: https://github.com/devonorourke/tidybug. A zip archive of the repo is available as well: https://doi.org/10.5281/zenodo.3929511. Individual database files are hosted at an Open Science Framework repo: https://osf.io/k3eh6. Raw sequence reads available at NCBI BioProject PRJNA518082: https://www.ncbi.nlm.nih.gov/bioproject/PRJNA518082
