# Supplementary figures and images for: A total crapshoot? Evaluating bioinformatic decisions in animal diet metabarcoding analyses
Source: Ecol Evol. 2020 Jul 23;10(18):9721–39. doi: 10.1002/ece3.6594 (PMC7520210; doi:10.1002/ece3.6594)

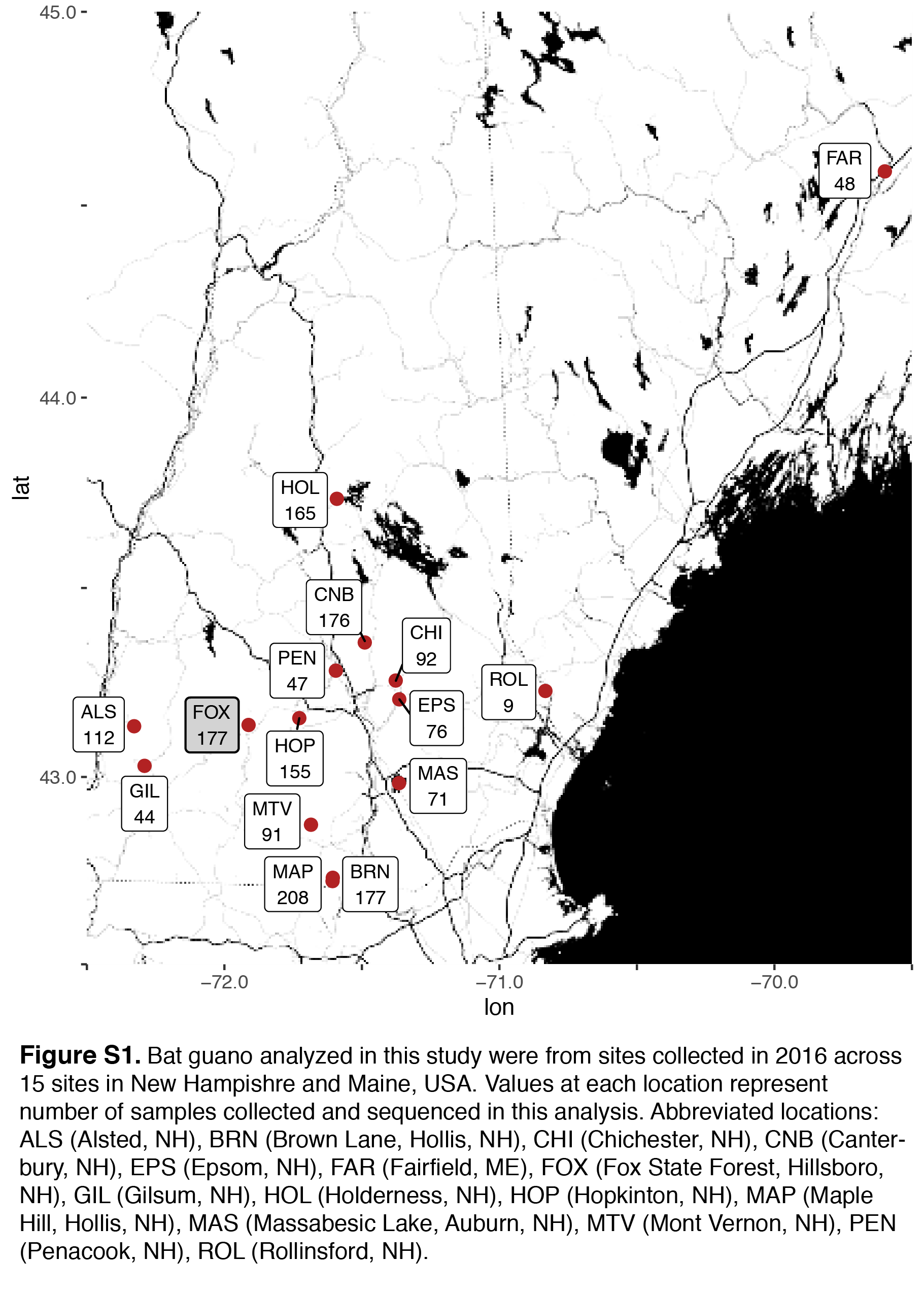

Supplement: Supplementary file 1 — Supplementary Material [file ECE3-10-9721-s001.zip › tidybug-master/SupplementaryFiguresTables/figureS1_collectionMap_withFigureLegend.png]

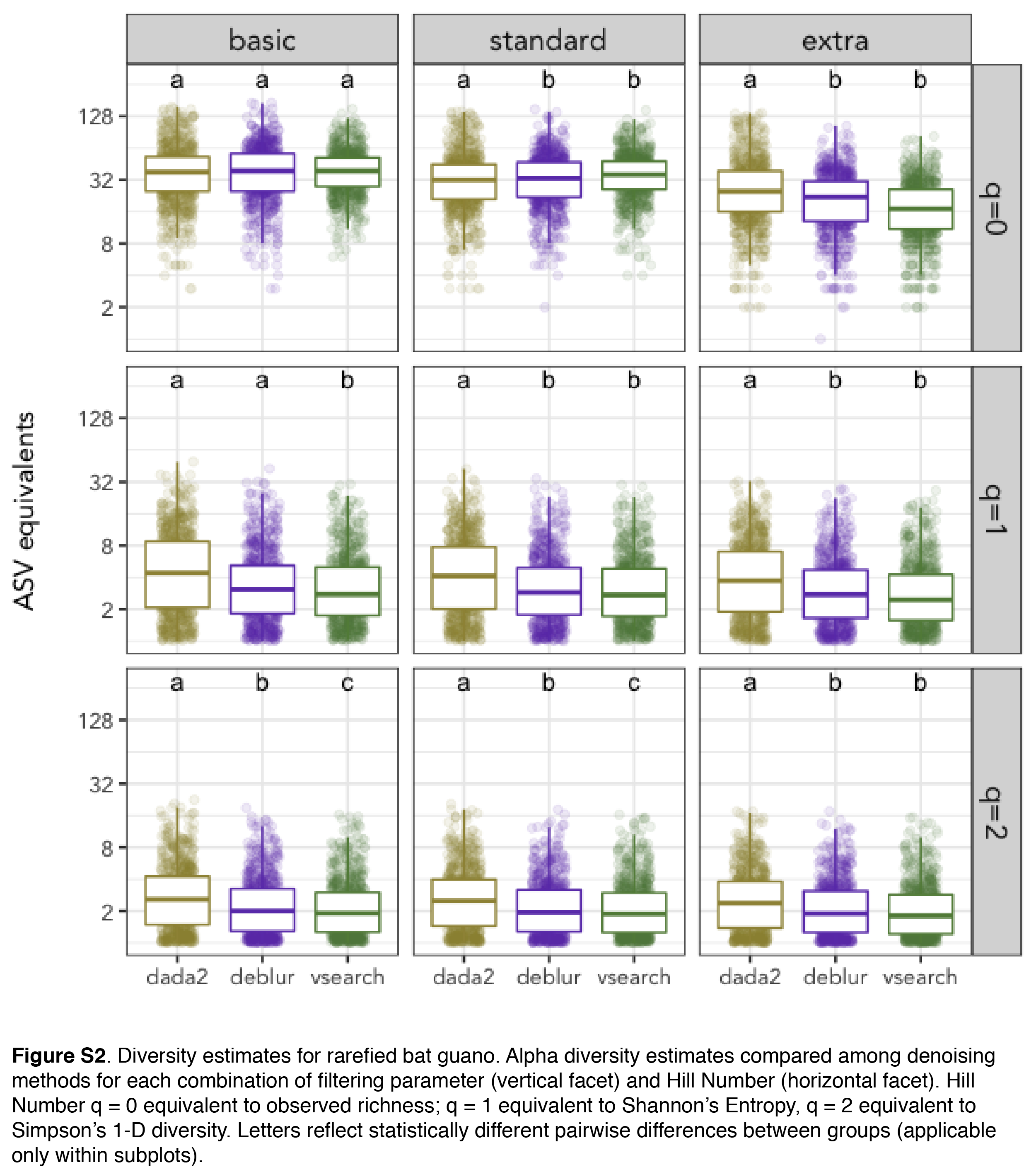

Supplement: Supplementary file 1 — Supplementary Material [file ECE3-10-9721-s001.zip › tidybug-master/SupplementaryFiguresTables/figureS2_guano_hillVals_wFigLegend.png]

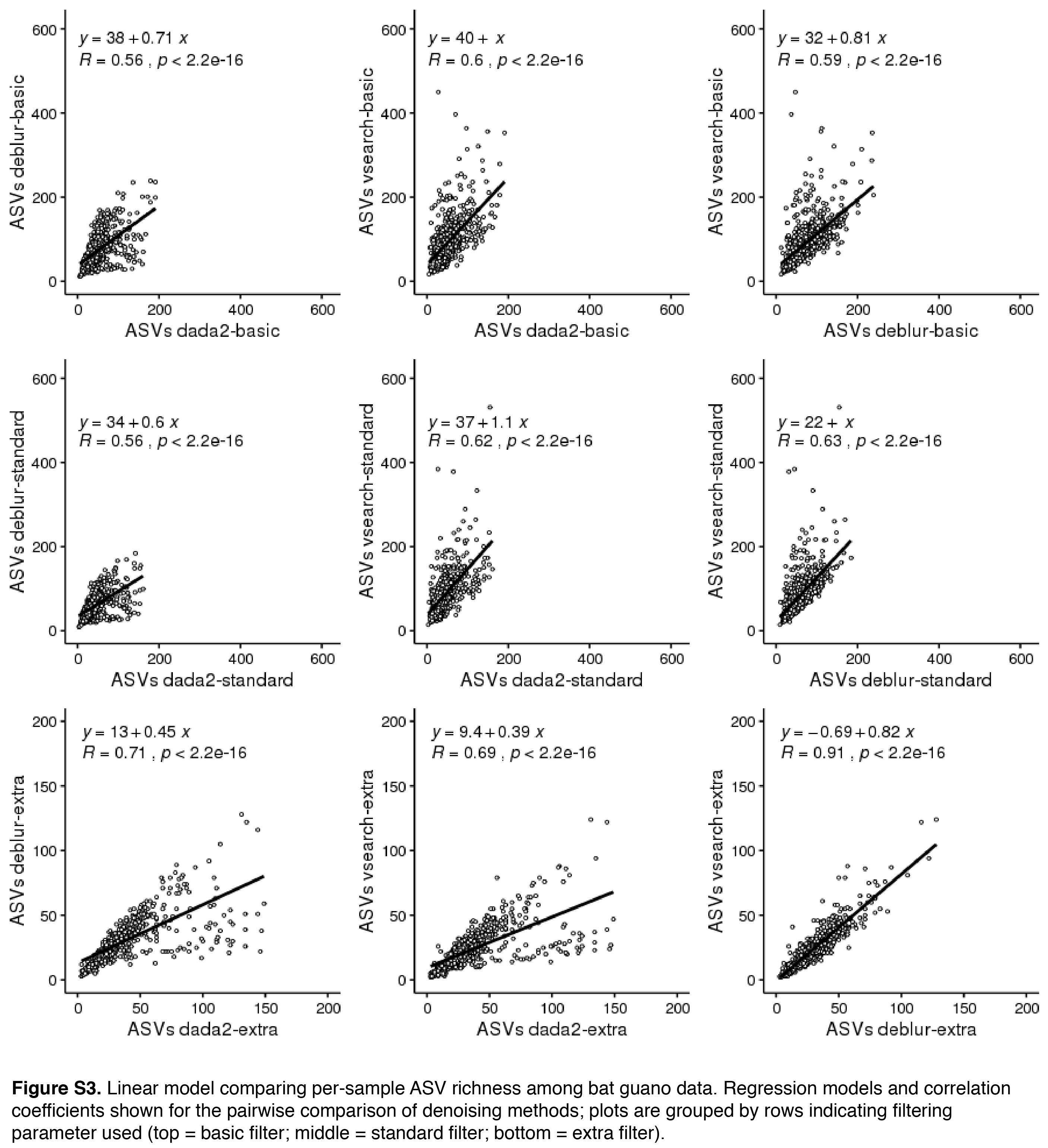

Supplement: Supplementary file 1 — Supplementary Material [file ECE3-10-9721-s001.zip › tidybug-master/SupplementaryFiguresTables/figureS3_asvRichness_correlation.png]

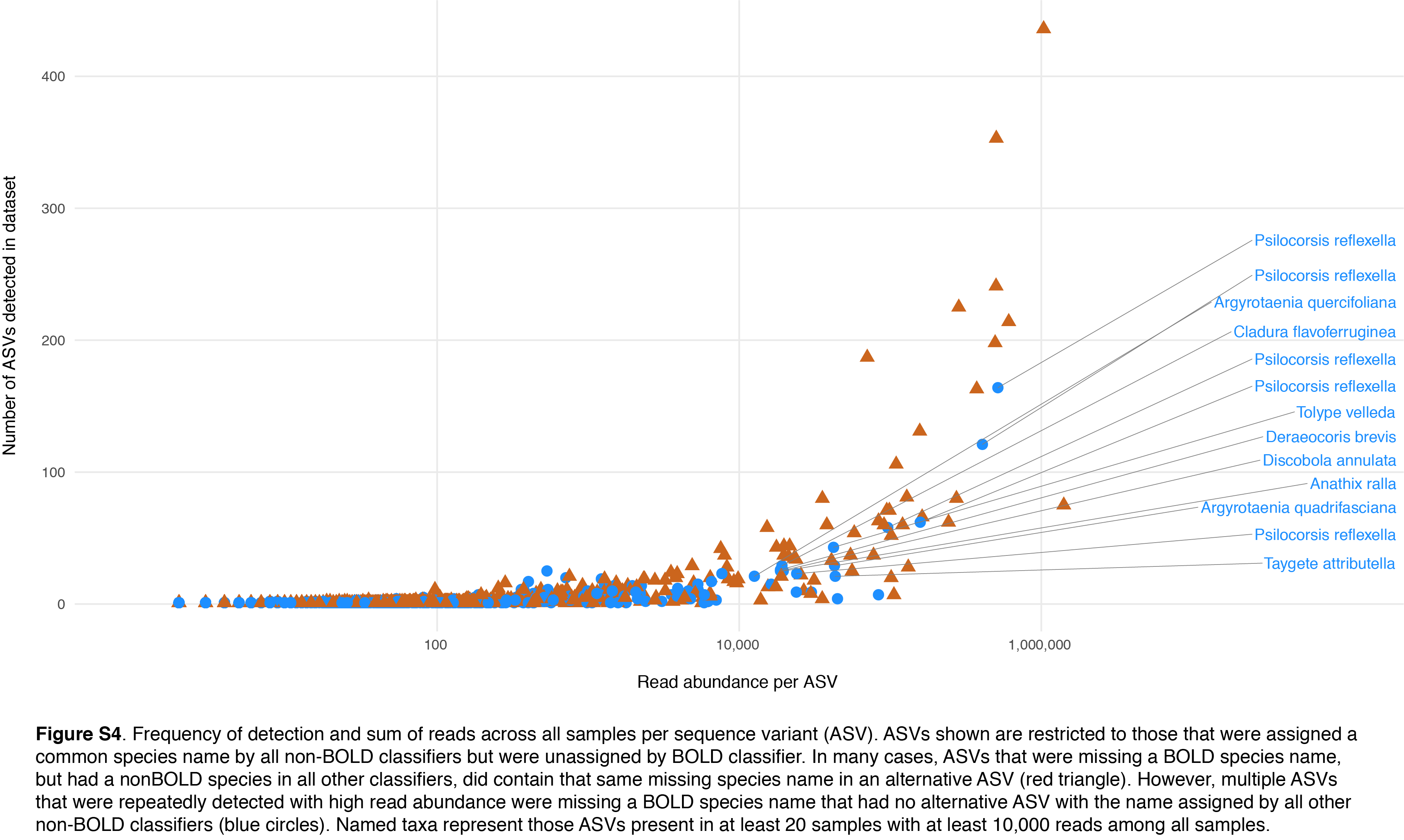

Supplement: Supplementary file 1 — Supplementary Material [file ECE3-10-9721-s001.zip › tidybug-master/SupplementaryFiguresTables/figureS4_classifier_consequence.png]

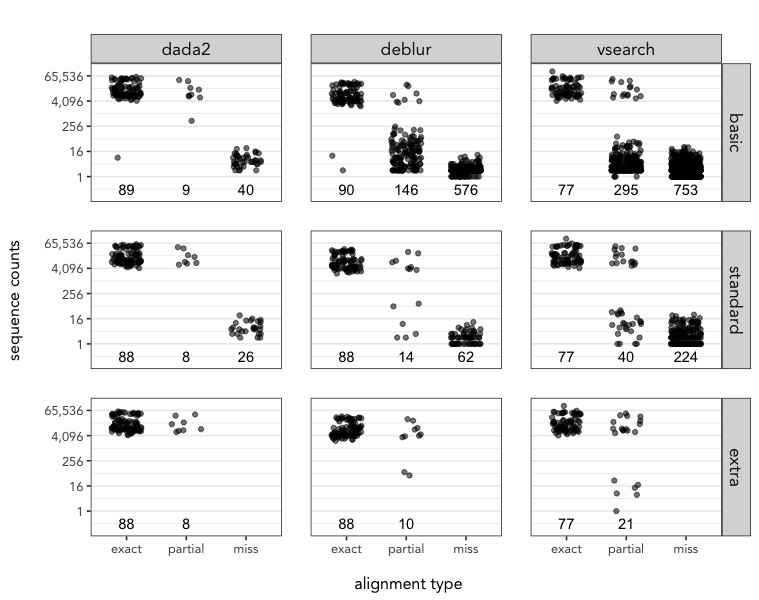

Supplement: Supplementary file 1 — Supplementary Material [file ECE3-10-9721-s001.zip › tidybug-master/figures/figure1_mockSeqs.png]

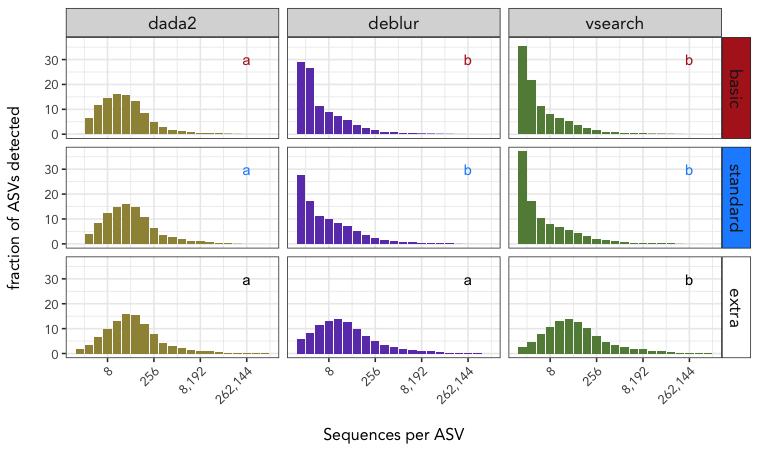

Supplement: Supplementary file 1 — Supplementary Material [file ECE3-10-9721-s001.zip › tidybug-master/figures/figure2_guanoHist.png]

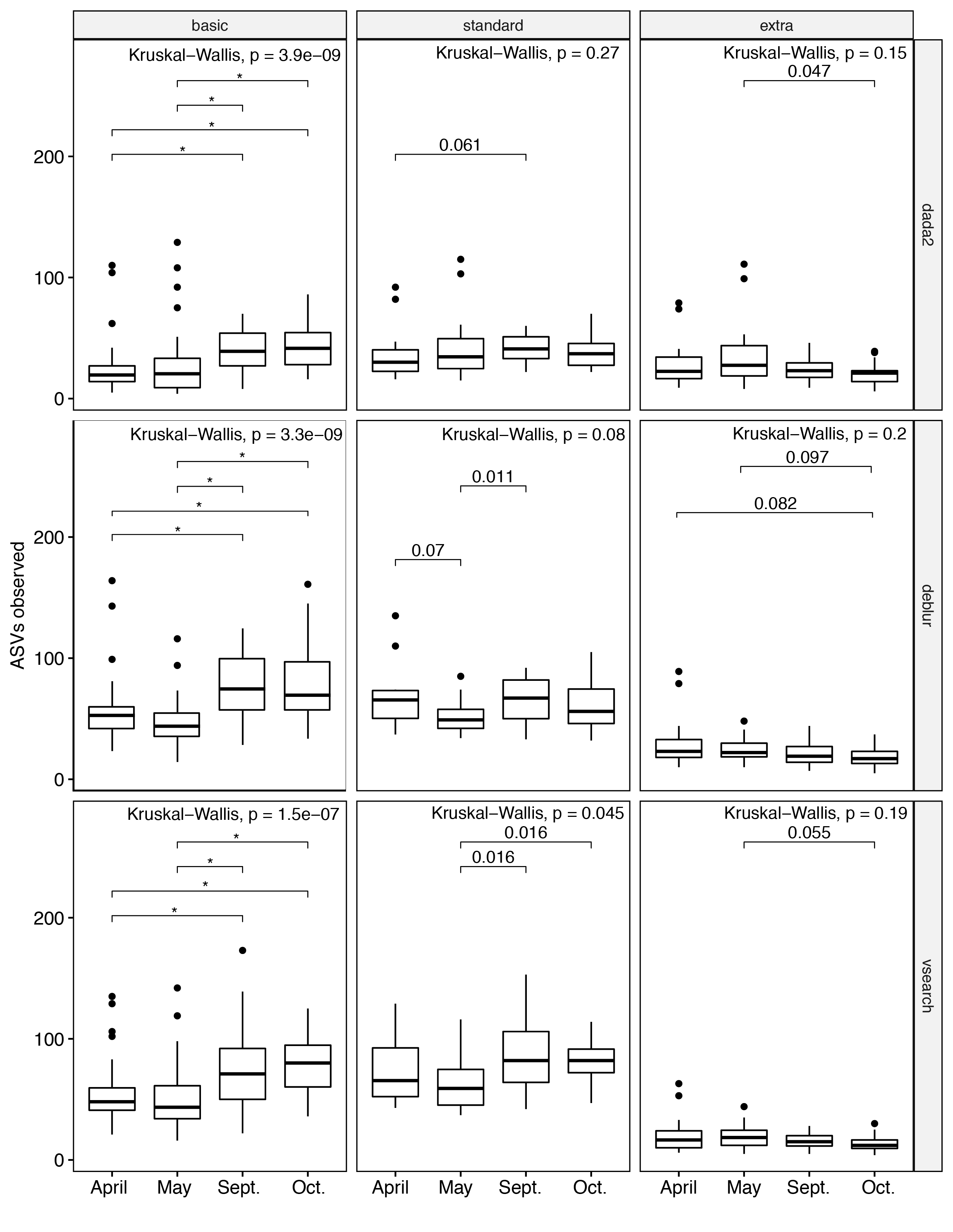

Supplement: Supplementary file 1 — Supplementary Material [file ECE3-10-9721-s001.zip › tidybug-master/figures/figure3_richnessComp_FoxData_manuallyUpdated.png]

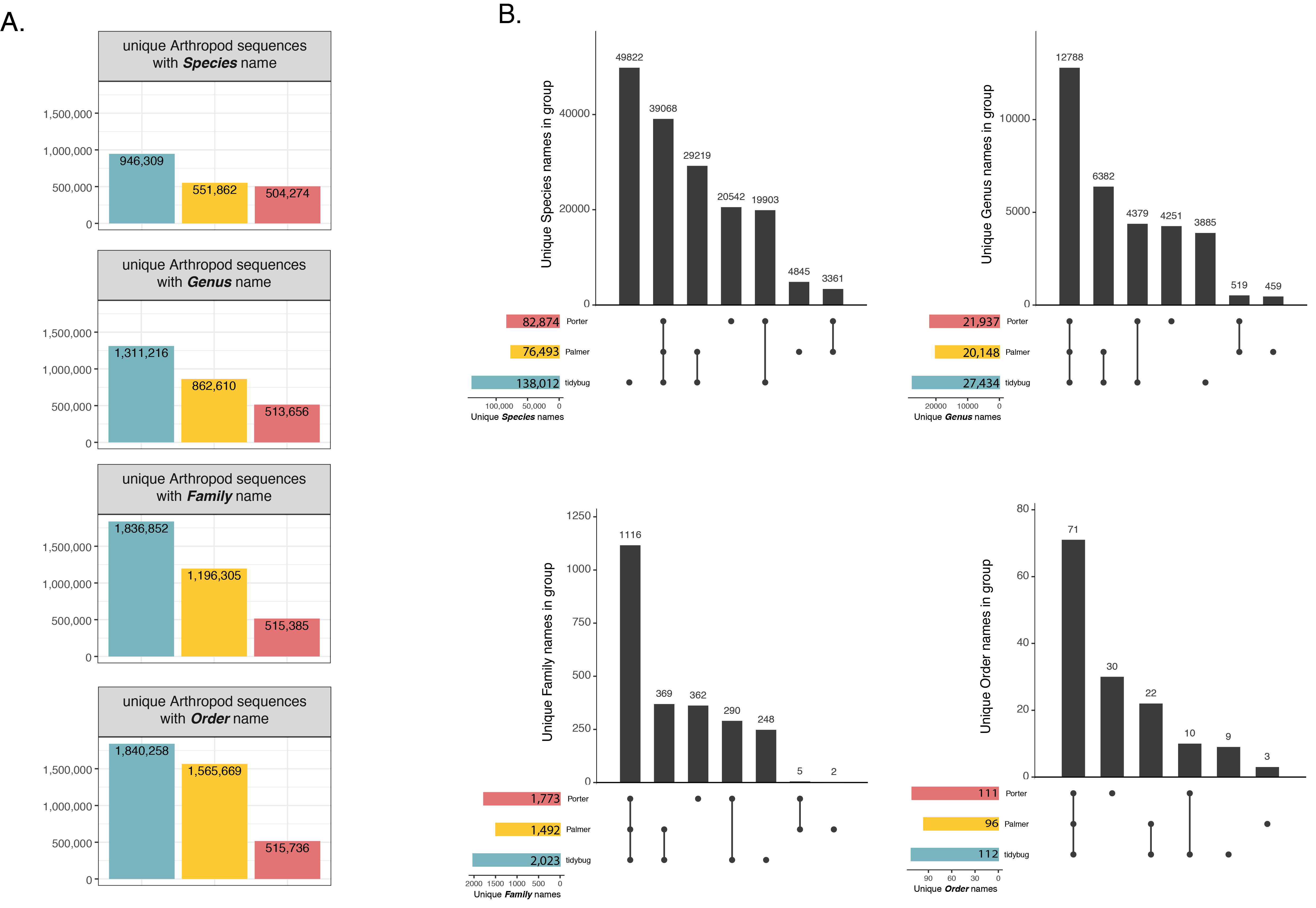

Supplement: Supplementary file 1 — Supplementary Material [file ECE3-10-9721-s001.zip › tidybug-master/figures/figure4_upsetPlot.png]

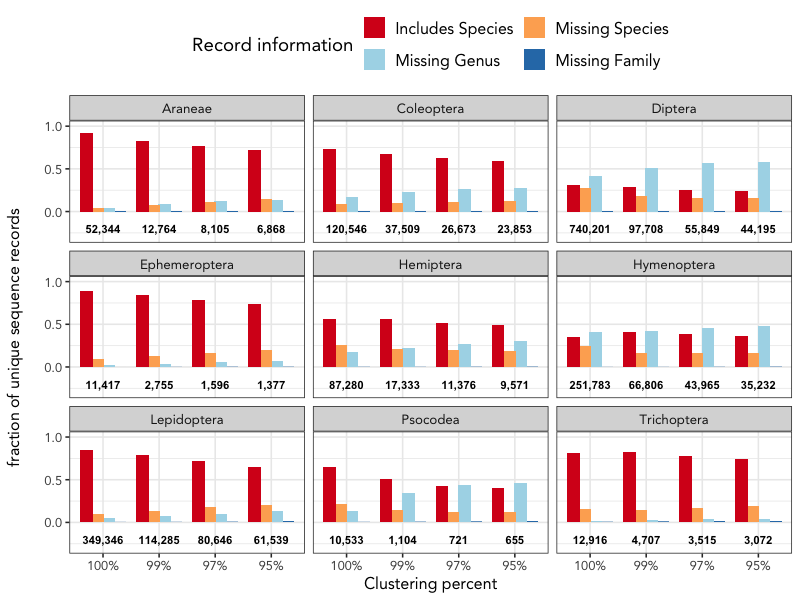

Supplement: Supplementary file 1 — Supplementary Material [file ECE3-10-9721-s001.zip › tidybug-master/figures/figure5_clustSelectOrders.png]

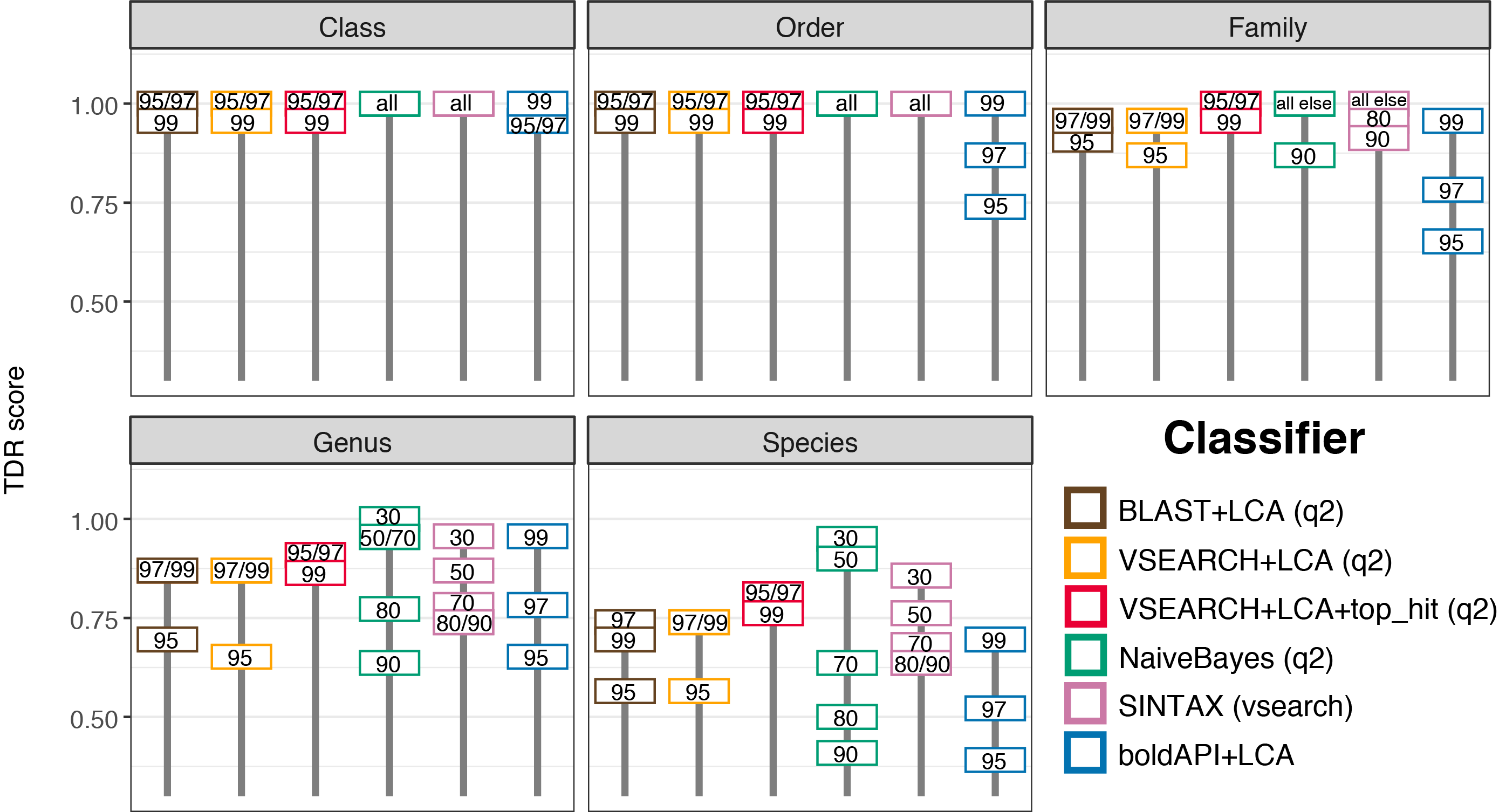

Supplement: Supplementary file 1 — Supplementary Material [file ECE3-10-9721-s001.zip › tidybug-master/figures/figure6_classifierComps_wVals.png]

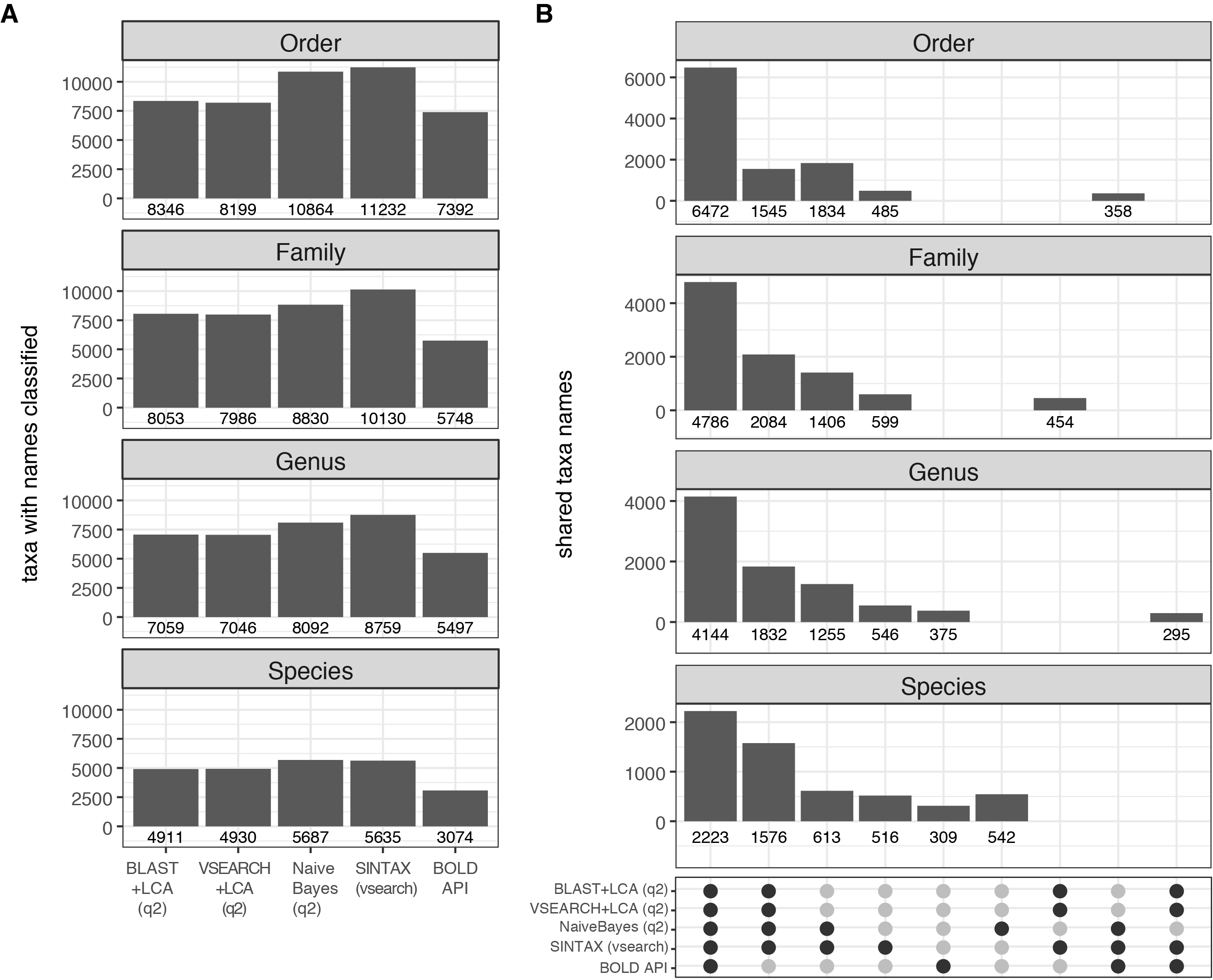

Supplement: Supplementary file 1 — Supplementary Material [file ECE3-10-9721-s001.zip › tidybug-master/figures/figure7_classifierComps.png]
